# Interdisciplinary CardioVascular and Thoracic Surgery—2022 Reviewers

**DOI:** 10.1093/icvts/ivad042

**Published:** 2023-03-09

On behalf of EACTS and EBCP, the Editor-in-Chief wishes to thank the following colleagues who have voluntarily given their valued time and effort to reviewing papers for ICVTS in 2022.

Kyomars Abbasi

Tomonobu Abe

Yukio Abe

Firas Abu Akar

Yasir Abu-Omar

Niv Ad

Iki Adachi

Prasad Adusumilli

E Andreas Agathos

Hitesh Agrawal

John Agzarian

Diana Aicher

Clemens Aigner

Koray Ak

Ahmet Ruchan Akar

Payam Akhyari

Zohair Al Halees

Nawwar Al-Attar

Johannes Albes

Maria Inmaculada Alcalde Mayayo

Ivan Aleksic

Cem Alhan

Jason Ali

Marco Alifano

Khaled Al-Kattan

Mohamad Alkhouli

Nelson Alphonso

Bahaaldin Alsoufi

Peter Alston

Nasser Altorki

Brunilda Alushi

Antonio Alvarez

Ignacio Amat-Santos

Marcello Ambrogi

Luca Ampollini

Olga Ananiadou

Kyriakos Anastasiadis

Nicholas Andersen

Robert Anderson

Rafael Andrade

Terezia Andrasi

Martin Andreas

Anders Andreasson

David Andrews

Eleni-Rosalina Andrinopoulou

Udo Anegg

Gianni Angelini

Dimitrios Angouras

Hendrik Ankersmit

Phillip Antippa

Manuel Antunes

Shabana Anwar

Jehangir Appoo

Beatrice Aramini

Arunkumar Arasappa

Claudia Arenz

Olgun Aribaş

Priyadharshanan Ariyaratnam

George Arnaoutakis

Rakesh Arora

Jose Arribas

Sinan Arsan

Gokhan Arslan

Panagiotis Artemiou

Tohru Asai

Boulos Asfour

Jalal Assouad

Eduardo Astrosa Martin

Thanos Athanasiou

Rizwan Attia

Tim Attmann

Andreas Auensen

John Augoustides

Erle Austin

Rüdiger Autschbach

Mohammad Azari

Anvar Baban

Mahesh Babu

Emile Bacha

Frank Baciewicz Jr.

Carl Backer

Catalin Badiu

Patrick Bagan

Nikolaos Baikoussis

Pietro Bajona

Robert Baker

Ihsan Bakir

Husam Balkhy

Ko Bando

Toru Bando

Vinayak Bapat

Fabio Barili

Giuseppe Barletta

Krzysztof Bartus

Mohammad Bashir

Hasan Batirel

Timm Bauer

Kathrin Bäumler

Victor Bautista-Hernandez

Yusuf Bayrak

Gwyn Beattie

Thomas Beaver

Erik Beckmann

Eric Bedard

Harinder Bedi

Jose Belda-Sanchis

Emre Belli

Umberto Benedetto

Leila Benhassen

Stefano Benussi

Elisabet Berastegui

Denis Berdajs

Mikolaj Berezowski

Tim Berger

Christian Bermudez

Luca Bertoglio

Pietro Bertoglio

Luca Bertolaccini

Charles Berul

Amit Bhargava

Fausto Biancari

Rocco Bilancia

Giuseppe Biondi-Zoccai

Gianluigi Bisleri

Mohamad Bittar

Henrik Bjursten

Sabine Bleiziffer

Percy Boateng

Antonio Bobbio

Simon Body

Lyubomyr Bohuta

Ciprian Bolca

Servet Bölükbas

Bruna Bolzan

Nikolaos Bonaros

Stefano Bongiolatti

Andreas Böning

Michael Borger

Jochen Börgermann

Mario Borin

Mimi Borrelli

Ahmed Boseila

Petre Botianu

Luca Botta

Tomaso Bottio

Ismail Bouhout

Wobbe Bouma

Thierry Bourguignon

Thierry Bove

Alexandros Briasoulis

Pierre-Yves Brichon

Christian Brizard

Alessandro Brunelli

Vito Bruno

Wolfgang Buhre

Marianna Buonocore

Gaetano Burriesci

Nicholas Burris

Jawad Butt

Lucio Cagini

Ufuk Cagirici

Antonio Calafiore

Alessio Campisi

Saida Campwala

Ayten Cangir

Christopher Cao

Filippo Capestro

Giovanni Carboni

Giuseppe Cardillo

Davide Carino

John Carr

Thierry Carrel

Angelo Carretta

Raymond Cartier

Edward Caruana

Vincenzo Caruso

Monica Casiraghi

Filip Casselman

Manuel Castella

Patrizio Castelli

Javier Castillo

Robert Cerfolio

Alfredo Cerillo

Laurens Ceulemans

David Chambers

John Chambers

Themistokles Chamogeorgakis

Yin-Kai Chao

Andrew Chatzis

Nilanjan Chaudhuri

Alexander Chausov

Massimo Chello

Raphaelle Chemtob

Cai Cheng

Yeung-Leung Cheng

Adnan Chhatriwalla

Giovanni Alfonso Chiariello

Sian Chivers

Torsten Christ

Michael Chu

Sertac Cicek

Paola Ciriaco

Joseph Clark

Julie Cleuziou

Andrea Colli

Miguel Congregado

Lenard Conradi

Pedro Correia

Joseph Coselli

Victor Costache

Garrett Coyan

Niccolo Daddi

Magnus Dalén

Vadim Dalinin

Thomas D'Amico

Mark Danton

John Dark

Gail Darling

Joao-Carlos Das-Neves-Pereira

Hiroshi Date

Hitendu Dave

Michele De Bonis

Laurent De Kerchove

Ruggero De Paulis

Raffaele De Simone

Filip De Somer

Joseph Dearani

Herbert Decaluwé

Georges Decker

Benedetto Del Forno

Pedro Del Nido

Victoria Delgado

Andrea Dell'Amore

Goran Dellgren

Philippe Demers

Stefanos Demertzis

Michelle Demetres

Onur Derdiyok

Manan Desai

Shriprasad Deshpande

Christian Detter

Frank Detterbeck

Anil Dharmapuram

Duccio Di Carlo

Marco Di Eusanio

Luca Di Marco

Michele Di Mauro

Giovanni Dialetto

Vanessa Diaz-Ravetllat

Arnaldo Dimagli

Ioannis Dimarakis

Zisis Dimitriadis

Xavier D'Journo

Torsten Doenst

Fabien Doguet

Daniel-Sebastian Dohle

Mary Donofrio

Augusto D'Onofrio

Mario Giovanni Gerardo D'Oria

Dimitrios Dougenis

Yves d'Udekem

John Duffy

Julia Dumfarth

Joel Dunning

Nandini Duraiswamy

Leonardo Duranti

Ahmet Durukan

Balthasar Eberle

Friedrich Eckstein

Jan Peter Eerenberg

Ahmed El-eshmawi

Aschraf El-Essawi

Haytham Elgharably

Stefano Elia

Sitaram Emani

Adelheid End

Shunsuke Endo

Abdullah Erdogan

Ersin Erek

Tahir Eren

Atilla Eroglu

Giampiero Esposito

Anthony Estrera

Gloria Faerber

Pierre-Emmanuel Falcoz

Francesco Faletra

Wentao Fang

Olivia Fanucchi

Fadi Farhat

Peter Feindt

Ioannis Felekos

Mark Ferguson

Elena Fernandez

Enrico Ferrari

Juan Fibla

Alfonso Fiorelli

Erwan Flecher

Nikolaos Floros

Sandra Folkmann

Laura Fong

Francesco Formica

Mahnoosh Foroughi

Christophoros Foroulis

Jose Fragata

Katrien Francois

Ulrich Franke

Jorge Freixinet

Stephen Fremes

Örjan Friberg

Ikuo Fukuda

Shinichi Fukuhara

Toshihiro Fukui

Satsuki Fukushima

Jozsef Furak

Mark Galantowicz

Domenico Galetta

Javier Gallego-Poveda

Filippo Tommaso Gallina

Michele Gallo

Carlos Galvez

Rafael Garcia-Fuster

Ovidio García-Villarreal

Hrvoje Gasparovic

Asmae Gassa

Mario Gaudino

James Gaynor

Cengiz Gebitekin

Hans Geissler

Gino Gerosa

Alessandro Giamberti

Alan Gillinov

Daniyar Gilmanov

Leonard Girardi

Marie-Noelle Giraud

Evaldas Girdauskas

Lara Girelli

Brian Glenville

David Glineur

Donald Glower

Heike Göbel

Wolfgang Goetz

Carmen Gomar

Alessandro Gonfiotti

Diego Gonzalez-Rivas

Arun Gopalakrishnan

Dominique Gossot

Roman Gottardi

Martin Grabenwöger

Felice Granato

Stuart Grant

Martin Grapow

Juan Grau

Valentina Grazioli

Michael Grimm

Kasper Grosen

William Grossi

Paul Gründeman

Jürg Grünenfelder

Dominik Guensch

Francesco Guerrera

Laura Chiara Guglielmetti

Robert Guidoin

Serdar Gunaydin

Jarmo Gunn

Lucile Gust

Rui Haddad

Jeffrey Hagen

Christoph Haller

Per Halvorsen

Abdel-Mohsen Hamad

Claude Hanet

Henrik Hansen

Jan Hinnerk Hansen

Emma Hansson

Amer Harky

Leanne Harling

Majid Harmouche

Wael Hassanein

Ali Can Hatemi

Hoda Hatoum

Ichiro Hayashi

David Healy

Claudia Heilmann

Markus Heinemann

Paul Heinisch

Khosro Hekmat

Olli Helminen

Marc Hendrikx

Mairead Hennessy

Samuel Heuts

Sven Hillinger

Dominique Himbert

Hirofumi Hioki

Sameer Hirji

Vibeke Hjortdal

Maurice Hogan

Johannes Holfeld

Tomas Holubec

Chung-Ping Hsu

Hung-Lung Hsu

Po-Kuei Hsu

Cheng-Long Huang

Narcis Hudorovic

Nabil Hussein

Mohsen Ibrahim

Hajime Ichikawa

Hitoshi Igai

Yasunori Iida

Shinichiro Ikeda

Ilkka Ilonen

Yoshito Inoue

Eveline Internullo

Keiichi Itatani

Krishna Iyer

Samuel Jacob

Oyvind Jakobsen

Erik Jansen

Katarzyna Januszewska

Omar Jarral

Eric Jeng

Marcelo Jimenez

Federica Jiritano

Ivan Joalsen

Richard Jonas

Guillaume Jondeau

David Jones

Wolfgang Jungraithmayr

Riyaz Kaba

Alexander Kadner

Nobuyuki Kagiyama

Larry Kaiser

David Kalfa

Klaus Kallenbach

Antonios Kallikourdis

Mohamed Kamel

Markus Kamler

Fikret Kanat

Emmanouil Kapetanakis

Tevfik Kaplan

Matthias Karck

Fabian Kari

Jamshid Karimov

Tom Karl

Riken Kawachi

Masafumi Kawamura

Aditya Kaza

Marlies Keijzers

Gunter Kerst

Suresh Keshavamurthy

Espeed Khoshbin

Arman Kilic

Doosang Kim

Hyung-Kwan Kim

Yong-Hee Kim

Naoyuki Kimura

Kaan Kirali

Bilal Kirmani

Attila Kiss

Soichiro Kitamura

Tadashi Kitamura

Thomas Kjeld

Robert Klautz

Jolanda Kluin

Walter Knirsch

Christoph Knosalla

Chris Knott-Craig

Gregor Kocher

Mladen Kocica

Luca Koechlin

Markus Kofler

Alexander Kogan

Philippe Kolh

Yu-Ichiro Koma

Stoyan Kondov

Igor Konstantinov

Tomislav Kopjar

Martin Kostolny

Christophoros Kotoulas

Marcin Kozuch

Markus Krane

George Krasopoulos

Maximilian Kreibich

Felix Kreidel

Bartosz Kubisa

Paul Kurlansky

Jaroslaw Kuzdzal

Young-Lan Kwak

Francois Lacour-Gayet

Tanel Laisaar

Ludwig Lampl

Patrizio Lancellotti

Michael Lanuti

Stephen Large

Robert Leatherby

Guillaume Lebreton

Jae Won Lee

Kyongjune Lee

Eric Lehr

Miia Lehtinen

David Leibowitz

Massimo Lemma

Toni Lerut

Mario Lescan

Gunda Leschber

Oliver Liakopoulos

Antonio Lio

Mauro Lo Rito

Antonio Loforte

Tom Loke

Jose Lopez-Menendez

Diego Lopez-Otero

Roberto Lorusso

Julian Losanoff

Attilio Lotto

Tsvetomir Louknaov

Gianluca Lucchese

Giovanni Battista Luciani

Heyman Luckraz

Maximilian Luehr

Michael Ma

Wei-Guo Ma

Guy Macgowan

Heinrich Mächler

Michael Mack

Francesco Maisano

Tarek Malas

Amber Malhotra

Norman Mangner

Tomohiro Maniwa

Yoshimasa Maniwa

Rita Marasco

Supreet Marathe

Stefano Margaritora

Mateo Marin-Cuartas

Alessio Mariolo

Andreas Martens

Thomas Martens

Carlos Martin Lopez

Parwis Massoudy

Stefano Mastrobuoni

Pasquale Mastroroberto

Munetaka Masuda

Pankaj Mathur

Hitoshi Matsuda

Haruhisa Matsuguma

Sandro Mattioli

Constantine Mavroudis

Alessandro Mazzucco

Elisa Meacci

Uwe Mehlhorn

Massimiliano Meineri

Philippe Menasche

Lorenzo Menicanti

Ari Mennander

Olaf Mercier

Frank Merkle

Carlos Mestres

Bart Meuris

Guido Michielon

Stephanie Mick

Marcello Migliore

Carmelo Mignosa

Tomislav Mihaljevic

Milan Milojevic

Kenji Minatoya

Shiro Miura

Kentaroh Miyoshi

Paul Modi

Daniela Molena

Thomas Molnar

Emilio Monguió

Luiz Moreira

Victor Morell

Paula Moreno

Tetsuro Morota

Marco Moscarelli

Victor Mosquera

Michael Mueller

Ludwig Müller

Giacomo Murana

Takashi Murashita

Piergiorgio Muriana

Diego Murillo Brito

John Murkin

Patrick Myers

Dania Nachira

Hani Najm

Masakazu Nakao

Francesco Nappi

Paolo Nardi

Antonio Nenna

Eugenio Neri

Christoph Nienaber

Thilo Noack

Nuria Novoa

Shahab Nozohoor

Satoshi Numata

Lauge Oestergaard

Hitoshi Ogino

Kenji Okada

Meinoshin Okumura

Guven Olgac

Burak Onan

Ismihan Onan

Geraldine Ong

Masamichi Ono

Francesco Onorati

Mohamed Osman

Brigitte Osswald

Murat Ozeren

Davide Pacini

Massimo Padalino

Ramdas Pai

Leonardo Paim

George Palatianos

Nikolaos Panagiotopoulos

Nikolaos Papakonstantinou

In Kyu Park

Kay-Hyun Park

Miralem Pasic

Francesco Patane

Kartik Patel

Gregory Pattakos

Syed Peer

Louis P Perrault

Bertrand Perrin

René Petersen

Sven Peterss

Mate Petricevic

Gosta Pettersson

Stephen Pettit

Joachim Photiadis

Gabriele Piffaretti

Hans Pilegaard

Clarence Pingpoh

Sergio Pirola

Jan Poelaert

Andrzej Polanczyk

Cecilia Pompili

Alain Poncelet

Shi Sum Poon

Zoran Popovic

Michael Poullis

Ourania Preventza

Anatol Prinzing

Elena Prisciandaro

Prakash Punjabi

Salah Qanadli

Eduard Quintana

Ehud Raanani

Shahbudin Rahimtoola

Mohamed Rahouma

Karthik Ramakrishnan

Marco Ranucci

Madhuri Rao

Sajjad Raza

Rajashekara Reddy

Shekar Reddy

Majed Refai

Diana Reser

Oliver Reuthebuch

Zaccaria Ricci

Mauro Rinaldi

Laura Rings

Marc Riquet

Gaetano Rocco

Albert Rodríguez-Fuster

Leonardo Roever

Luke Rogers

Sonia Ronchey

Olena Rudyk

André Rüffer

Dieuwertje Ruigrok

Bartosz Rylski

Michel Pompeu Sá

Satoshi Saito

Tomoki Sakata

Shoji Sakiyama

Mohamed Salama

Antonio Salsano

Sacha Salzberg

Joao Santos Silva

Nishant Saran

Ulrik Sartipy

Milton Saute

Yoshiki Sawa

Noriyoshi Sawabata

Paolo Scanagatta

Thomas Schachner

Hartzell Schaff

Michael Scharfschwerdt

Stefano Schena

David Schibilsky

Marc Schindewolf

Georg Schlachtenberger

Markus Schlömicher

Ralph Schmid

Jürg Schmidli

Florian Schoenhoff

Paul Schoenhogen

Richard Schuessler

Leon Schurgers

Thomas Schwann

Michael Seco

Moritz Seiffert

Massimo Sella

Daniel Sellers

Paul Sergeant

Michael Shackcloth

Mohammad Shadmehr

Baoen Shan

Yaron Shargall

Rajesh Sharma

Mikalai Shchatsinka

Sharaf-Eldin Shehada

William Shi

Norihisa Shigemura

Norihiko Shiiya

Yasushige Shingu

Yuji Shiraishi

Yukitoshi Shirakawa

Malakh Shrestha

Dominique Shum-Tim

Sailay Siddiqi

Jacobo Silva

Pranava Sinha

Thanos Sioris

Muammer Sivrikoz

Francis Smit

Freyja Smolle-Jüttner

Thomas Snow

Laura Socci

Sebastian-Patrick Sommer

Bong Gun Song

Howard Song

Miguel Sousa-Uva

Lorenzo Spaggiari

Jan Spillner

Stefan Sponholz

Chris Spurney

Mila Stajevic

Alessia Stanzi

Christoph Starck

Alessandro Stefani

Brigitte Stiller

Serban Stoica

Thoralf Sundt

Staffan Svenmarker

Ben Swinkels

Sarah Tabbutt

Federico Tacconi

Tamaki Takano

Sachin Talwar

Derrick Tam

Aikaterini Christina Tampaki

Tomoko Tani

James Tatoulis

Adele Tessitore

Stefan Thelin

Kassiani Theodoraki

Pascal Thomas

Matthieu Thumerel

Tomasz Timek

Kaushal Tiwari

Anton Tomsic

Aybala Tongut

Davide Tosi

Francesca Toto

Piergiorgio Tozzi

Nikolay Travin

Tom Treasure

Santi Trimarchi

Georg Trummer

Konstantinos Tsagakis

Victor Tsang

Elaine Tseng

Nikolaos Tsilimparis

Marko Turina

James Tweddell

Agnieszka Tycinska

Paula Ugalde Figueroa

Murat Ugurlucan

Victor Umans

Philippe Unger

Glen Van Arsdel

Eric Van Belle

Andreas Van De Loo

Caroline Van De Wauwer

Karel Van Praet

Bart Van Putte

Dirk Van Raemdonck

Paul Van Schil

Hans Van Veer

Jacopo Vannucci

Panos Vardas

Gonzalo Varela

Giuseppe Vassalli

Prem Venugopal

Peter Verbrugghe

Giulia Veronesi

Dominique Vervoort

Emmanuel Villa

Hunaid Vohra

Pierre Voisine

Luca Voltolini

Konstantin Von Aspern

Yskert von Kodolitsch

Ulrich von Oppell

Alain Vuylsteke

Andrew Waberski

Alexander Wahba

Thorsten Walles

Isaac Wamala

Bangmao Wang

Shuwei Wang

Atsushi Watanabe

Taiju Watanabe

Yui Watanabe

Stefan Watzka

Alberto Weber

Katrin Welcker

Luca Weltert

Paul Werner

Gil Wernovsky

Douglas West

Chris Whight

Dominik Wiedemann

Gerhard Wimmer-Greinecker

Felix Woitek

Randolph Wong

Ming-Ho Wu

Qinzi Xu

Yoshitaka Yamane

Mohammed Yasin

Can Yerebakan

Bedrettin Yildizeli

Kanhua Yin

Amir Youssari

Joseph Zacharias

Mustafa Zakkar

Vipin Zamvar

Edoardo Zancanaro

Francesco Zaraca

Jun-Ming Zhu

Alicja Zientara

Peter Zilla

Daniel Zimpfer

Charalambos Zisis

Andrea Zuin

